# Machine learning-based risk factor analysis of necrotizing enterocolitis in very low birth weight infants

**DOI:** 10.1038/s41598-022-25746-6

**Published:** 2022-12-10

**Authors:** Hannah Cho, Eun Hee Lee, Kwang-Sig Lee, Ju Sun Heo

**Affiliations:** 1 Department of Pediatrics, Korea University College of Medicine, Anam Hospital, 73 Goryeodae-Ro, Seongbuk-Gu, Seoul, 02841 Korea; 2 Department of Pediatrics, Korea University Anam Hospital, Seoul, Korea; 3 AI Center, Korea University College of Medicine, Anam Hospital, 73 Goryeodae-Ro, Seongbuk-Gu, Seoul, 02841 Korea

**Keywords:** Environmental sciences, Diseases, Risk factors

## Abstract

This study used machine learning and a national prospective cohort registry database to analyze the major risk factors of necrotizing enterocolitis (NEC) in very low birth weight (VLBW) infants, including environmental factors. The data consisted of 10,353 VLBW infants from the Korean Neonatal Network database from January 2013 to December 2017. The dependent variable was NEC. Seventy-four predictors, including ambient temperature and particulate matter, were included. An artificial neural network, decision tree, logistic regression, naïve Bayes, random forest, and support vector machine were used to evaluate the major predictors of NEC. Among the six prediction models, logistic regression and random forest had the best performance (accuracy: 0.93 and 0.93, area under the receiver-operating-characteristic curve: 0.73 and 0.72, respectively). According to random forest variable importance, major predictors of NEC were birth weight, birth weight Z-score, maternal age, gestational age, average birth year temperature, birth year, minimum birth year temperature, maximum birth year temperature, sepsis, and male sex. To the best of our knowledge, the performance of random forest in this study was among the highest in this line of research. NEC is strongly associated with ambient birth year temperature, as well as maternal and neonatal predictors.

## Introduction

Necrotizing enterocolitis (NEC) occurs in 5–10% of very low birth weight (VLBW) infants and is one of the leading causes of death among them^1–3^. It is known that even survivors of NEC eventually come down with long-term growth failure and neurodevelopmental impairments^4–7^.

The pathogenesis of the clinical entity known as NEC is multifactorial. Traditionally, immaturity, hyperosmolar formula, fast feeding advance, infection, and bowel ischemia are known risk factors for NEC^8–14^. In addition, several studies have investigated the association between seasonal variations and NEC^15–18^. A previous multicenter study in the US showed a biphasic high peak occurrence of NEC in May/June and October/November^15^. Javidi et al. reported a similar bimodal peak and higher number of NEC in April/May^16^. In this study, gestational age (GA), birth weight (BW), and birth month were associated with NEC. Another multicenter study in England showed that the incidence of surgical NEC was higher in late spring ^17^. However, a study in Sweden found a peak incidence in November and a low incidence in May^18^. These studies, though with inconsistent results, revealed that environmental factors such as seasonal variation and birth month could influence the incidence of NEC. Studies on the association between NEC and other environmental factors, such as ambient temperature and air pollution are lacking. Furthermore, no endeavors have been made regarding the utilization of machine learning for the prediction of NEC among VLBW infants.

In this context, this study employed machine learning and a national prospective cohort registry database to examine the main predictors of NEC in VLBW infants, including environmental factors such as ambient temperature, air pollution, and seasonal variation in birth year. This study presents the most comprehensive machine learning analysis on this topic, using a rich collection of 74 predictors and bringing new results concerning their associations with NEC.

## Results

Descriptive statistics for NEC and its categorical predictors are presented in Table 1. Among 10,353 VLBW infants, the proportion of NEC was 6.8% (n = 704). The results of the univariate analysis (chi-square test for the equality of proportions “Yes” or t test for the equality of means) are presented in Table 2. The *P* values were smaller than 0.05 for the following variables: GA, BW, small-for-GA, sex (male), birth year, multipara, gestational diabetes mellitus, chorioamnionitis, pre-labor rupture of membrane, antenatal steroid use, cesarean section, oligohydramnios, polyhydramnios, Apgar score, intensive neonatal resuscitation, initial blood gas analysis, pulmonary hemorrhage, respiratory distress syndrome, treated patent ductus arteriosus (PDA), air leak syndrome, and sepsis.

**Table 1:** Descriptive Statistics: Necrotizing Enterocolitis and Categorical Predictors.

Variable	*n*	*%*
Necrotizing enterocolitis	704	6.8
Gestational age < 28 weeks	3902	37.7
Gestational age < 26 weeks	1874	18.1
Birthweight < 1000 g	3900	37.7
Birthweight < 750 g	1611	15.6
Small-for-gestational-age	2224	21.6
Sex: male	5115	49.4
**Birth-Year**		
2013	1392	13.4
2014	2119	20.5
2015	2380	23.0
2016	2346	22.7
2017	2116	20.4
Birth-Season: Spring	2513	24.3
Birth-Season: Summer	2608	25.2
Birth-Season: Autumn	2738	26.4
Birth-Season: Winter	2494	24.1
Multiple pregnancy	3637	35.1
Multipara	6448	62.3
In vitro fertilization	2389	23.1
Gestational DM	826	8.0
Overt DM	114	1.1
Pregnancy-induced hypertension	1976	19.1
Chronic hypertension	221	2.1
Chorioamnionitis	2989/8612	34.7
PROM > 18 h	2455/10,266	23.9
Antenatal steroid	8071/10,162	79.4
Cesarean section	8052	77.8
Oligohydramnios	1399/9442	14.8
Polyhydramnios	153/9442	1.6
Congenital infection	127	1.2
1-min Apgar score ≤ 3	2975/10,278	28.9
5-min Apgar score < 7	3488/10,283	33.9
Neonatal resuscitation program	9169/10,281	89.2
Neonatal resuscitation program intensive	6466/10,281	62.9
Blood gas pH < 7.0	503/7792	6.5
Blood gas base excess < -15	235/7768	3.0
Pulmonary hemorrhage	636	6.1
Respiratory distress syndrome	8058	77.8
Surfactant count ≥ 2	2081	20.1
Patent ductus arteriosus treatment	3705/10,044	36.9
Patent ductus arteriosus ligation	1099/7296	15.1
Air leak syndrome	569	5.5
Sepsis	2177	21.1

Abbreviations: DM, Diabetes mellitus; PROM, Pre-labor rupture of membrane.

**Table 2:** Univariate Analysis.

Variable	Necrotizing enterocolitis		
	No (n = 9649)	Yes (n = 704)	*P* value
Gestational age < 28 weeks	3412 (35.4)	490 (69.6)	< 0.001*
Gestational age < 26 weeks	1582 (16.4)	292 (41.5)	< 0.001*
Birth weight < 1000 g	3413 (35.4)	487 (69.2)	< 0.001*
Birth weight < 750 g	1360 (14.1)	251 (35.7)	< 0.001*
Small-for-gestational-age	2103 (21.9)	121 (17.4)	0.008*
Sex (male)	4852 (50.3)	386 (54.8)	0.048*
Birth-Year, n (%)			0.017*
2013	1290 (13.4)	102 (14.5)	
2014	1988 (20.6)	131 (18.6)	
2015	2234 (23.2)	146 (20.7)	
2016	2173 (22.5)	173 (24.6)	
2017	1964 (20.4)	152 (21.6)	
Birth-Season: Spring	2339 (24.2)	174 (24.7)	0.365
Birth-Season: Summer	2422 (25.2)	186 (26.4)	0.569
Birth-Season: Autumn	2549 (26.4)	189 (26.8)	0.774
Birth-Season: Winter	2339 (24.2)	155 (22.2)	0.164
Temperature average Year	14.14 ± 0.62	14.15 ± 0.64	0.654
Temperature minimum Year	8.87 ± 0.84	8.90 ± 0.87	0.446
Temperature maximum Year	20.22 ± 0.50	20.21 ± 0.52	0.788
PM_10_ Year	47.09 ± 0.59	47.11 ± 0.57	0.301
PM_10_ 10-month before birth	46.28 ± 9.13	46.66 ± 9.55	0.307
PM_10_ 09-month before birth	46.52 ± 9.20	46.13 ± 8.94	0.265
PM_10_ 08-month before birth	46.77 ± 9.32	46.90 ± 9.58	0.728
PM_10_ 07-month before birth	47.07 ± 9.40	46.93 ± 9.35	0.702
PM_10_ 06-month before birth	46.86 ± 9.24	46.94 ± 9.48	0.829
PM_10_ 05-month before birth	46.66 ± 9.35	46.04 ± 8.94	0.077
PM_10_ 04-month before birth	46.38 ± 9.40	46.05 ± 9.41	0.369
PM_10_ 03-month before birth	46.12 ± 9.51	46.12 ± 9.61	1.000
PM_10_ 02-month before birth	45.97 ± 9.42	46.47 ± 9.64	0.184
PM_10_ 01-month before birth	45.98 ± 9.33	45.99 ± 8.96	0.977
PM_10_ 00-month before birth	46.17 ± 9.32	46.54 ± 9.24	0.306
Multiple pregnancy	3412 (35.4)	225 (32.0)	0.188
Multipara	3614 (37.5)	291 (41.3)	0.050*
In vitro fertilization	2245 (23.3)	144 (20.5)	0.192
Gestational DM	794 (8.2)	32 (4.5)	0.002*
Overt DM	108 (1.1)	6 (0.9)	0.546
Pregnancy-induced hypertension	1859 (19.3)	114(16.6)	0.134
Chronic hypertension	205 (2.1)	16 (2.3)	0.886
Chorioamnionitis	2765 (28.7)	224 (31.8)	0.001*
PROM > 18 h	2273 (23.6)	182 (25.9)	< 0.001*
Antenatal steroid	7507 (77.8)	564 (80.1)	< 0.001*
Cesarean section	7545 (78.2)	507 (72)	0.001*
Oligohydramnios	1309 (13.6)	90 (12.8)	< 0.001*
Polyhydramnios	144 (1.5)	9 (1.3)	0.001*
Congenital infection	115 (1.2)	12(1.7)	0.485
1-min Apgar score ≤ 3	2667 (27.6)	308 (43.8)	< 0.001*
5-min Apgar score < 7	3142 (32.6)	346 (49.1)	< 0.001*
Neonatal resuscitation program	8508 (88.2)	661 (93.9)	< 0.001*
Neonatal resuscitation program, intensive	5888 (61.0)	578(82.1)	< 0.001*
Blood gas pH < 7.0	210 (2.2)	24 (3.4)	< 0.001*
Blood gas base excess < -15	205 (2.1)	30 (4.3)	< 0.001*
Pulmonary hemorrhage	532 (5.5)	104 (14.8)	< 0.001*
Respiratory distress syndrome	7414 (76.8)	644 (91.5)	< 0.001*
Surfactant use ≥ 2	1860 (19.3)	221 (31.4)	< 0.001*
Patent ductus arteriosus treatment	3348 (34.7)	357 (50.7)	< 0.001*
Patent ductus arteriosus ligation	934 (9.7)	165 (23.4)	< 0.001*
Air leak syndrome	485 (5.0)	84 (11.9)	< 0.001*
Sepsis	1854 (19.2)	323 (45.9)	< 0.001*

Values are presented as number (%) or mean ± standard deviation.

Abbreviations: DM, Diabetes mellitus; PROM, Pre-labor rupture of membrane; PM_10_, particulate matter concentration.

*P < 0.05 Chi-Square Test for the Equality of Proportions “Yes” or T Test for the Equality of Means.

**Figure 1:**
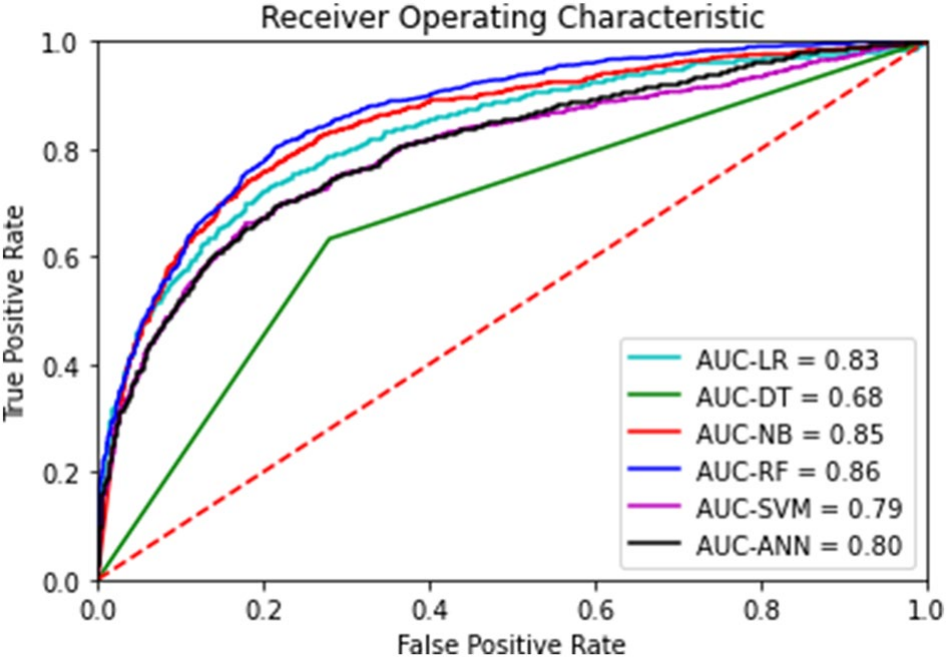
Area Under the Receiver-Operating-Characteristic Curves for Necrotizing Enterocolitis. Legend: The area under the receiver-operating-characteristic curve (AUC) is the plot of the true positive rate (sensitivity) against the false positive rate (1- specificity) at various threshold settings. Abbreviations: ANN Artificial neural network, AUC Area under the receiver-operating-characteristic curve, DT Decision tree, LR Logistic regression, NB Naïve Bayes, RF Random forest, SVM Support vector machine.

**Figure 2:**
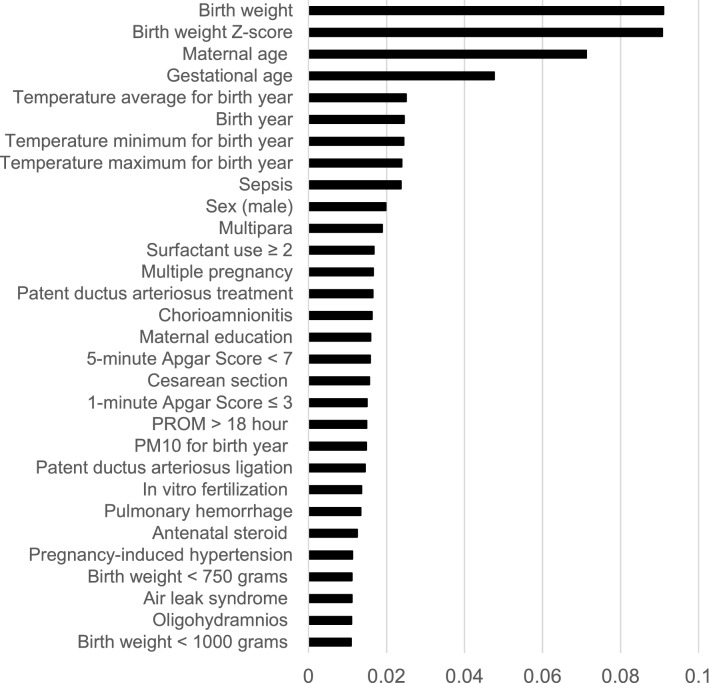
Random Forest Variable Importance Plots for Necrotizing Enterocolitis. Legend: Random forest variable importance calculates node impurity (GINI) decrease from the creation of a branch on a certain predictor. It is an average over all trees in a random forest with the range of 0 and 1. Abbreviations: PM, Particulate matter; PROM, Pre-labor rupture of membranes.

The performance measures for the six prediction models for NEC are listed in Table 3. The random split and analysis were repeated 50 times then its average was taken for external validation. The performance results were similar, irrespective of the inclusion of average ambient temperature for each of the 10, 9, 8, …, 2, 1, and 0 months before birth. With the inclusion of sepsis, the area under the receiver-operating-characteristic curve for the random forest increased from 0.70 to 0.72. Among the six prediction models for NEC, logistic regression and the random forest with 1000 trees had the best performance (accuracy: 0.93 and 0.93, area under the receiver-operating-characteristic curve: 0.73 and 0.72, respectively). The findings of hyper-parameter tuning in the last box of Table 3 show that the random forests with 500, 400, 300, 200 and 100 trees were not as good as the random forest with 1000 trees. Indeed, the area under the receiver-operating-characteristic curves for the six prediction models in one of the 50 runs are presented in Fig. 1. The results in Fig. 1 came from one particular run (i.e., the 50^th^ run), whereas the results in Table 3 are the averages of the 50 runs. This explains why they are different from each other. The values and ranks of random forest variable importance are presented in Table 4. The importance rank of the temperature average for each of the 10, 9, 8, …, 2, 1, and 0 months before birth was below the top 30, while their sepsis counterparts were within the top 10 (9th). According to the random forest variable importance in Table 4 and Fig. 2, the major predictors of NEC were BW (0.0910), BW Z-score (0.0907), maternal age (0.0712), GA (0.0476), average birth year temperature (0.0250), birth year (0.0245), minimum birth year temperature (0.0244), maximum birth year temperature (0.0239), sepsis (0.0237), sex (male) (0.0198), multipara (0.0189), surfactant use ≥ 2 (0.0168), multiple pregnancy (0.0166), treated PDA (0.0165), and chorioamnionitis (0.0163). Based on logistic regression variable importance (the absolute value of the optimized coefficient) in Table 5, indeed, major predictors of NEC were sepsis, BW Z-score, gestational diabetes mellitus, PDA ligation, unmarried, pulmonary hemorrhage, sex (male), maximum birth year temperature, air leak syndrome, chorioamnionitis, small-for-GA, blood gas base excess, GA, in vitro fertilization, and antenatal steroid. It needs to be noted that the results in Tables 4 and 5 came from one particular run (i.e., the 50th run).

**Table 3:** Model performance for predicting necrotizing enterocolitis: Means and confidence intervals over 50 runs.

	Accuracy			AUC		
Model	Mean	CI-L	CI-U	Mean	CI-L	CI-U
LR	0.933	0.931	0.933	0.722	0.715	0.722
DT	0.866	0.864	0.866	0.529	0.524	0.529
NB	0.737	0.733	0.737	0.708	0.702	0.708
RF-1000	0.932	0.930	0.932	0.702	0.696	0.702
SVM	0.933	0.931	0.933	0.404	0.395	0.404
ANN-10	0.911	0.909	0.911	0.714	0.707	0.714

*TABM*	Accuracy			AUC		
Model	Mean	CI-L	CI-U	Mean	CI-L	CI-U
LR	0.931	0.929	0.931	0.725	0.719	0.725
DT	0.866	0.864	0.866	0.526	0.522	0.526
NB	0.739	0.735	0.739	0.705	0.697	0.705
RF-1000	0.930	0.929	0.930	0.701	0.694	0.701
SVM	0.931	0.929	0.931	0.431	0.423	0.431
ANN-10	0.933	0.931	0.933	0.500	0.500	0.500

*TABM-S*	Accuracy			AUC		
Model	Mean	CI-L	CI-U	Mean	CI-L	CI-U
LR	0.932	0.931	0.932	0.730	0.723	0.730
DT	0.869	0.867	0.869	0.536	0.531	0.536
NB	0.737	0.732	0.737	0.721	0.715	0.721
RF-1000	0.932	0.931	0.932	0.724	0.716	0.724
SVM	0.932	0.930	0.932	0.340	0.331	0.340
ANN-10	0.932	0.931	0.932	0.698	0.690	0.698

*TUNING*	Accuracy			AUC		
Model	Mean	CI-L	CI-U	Mean	CI-L	CI-U
RF-500	0.932	0.931	0.932	0.718	0.716	0.724
RF-400	0.933	0.931	0.933	0.716	0.711	0.716
RF-300	0.933	0.932	0.933	0.717	0.712	0.717
RF-200	0.930	0.928	0.930	0.712	0.708	0.712
RF-100	0.933	0.932	0.933	0.717	0.711	0.717
ANN-20	0.932	0.930	0.932	0.500	0.500	0.500

Abbreviations: ANN Artificial neural network, AUC Area under the receiver-operating-characteristic curve, CI-L Lower bound of 95% confidence interval, CI-U Upper bound of 95% confidence interval, DT Decision tree, LR Logistic regression, NB Naïve Bayes, RF Random forest, SVM Support vector machine, TABM 3 variables (ambient temperature average, minimum and maximum for birth month in Table 2) included, TABM-S 4 variables (ambient temperature average, minimum and maximum for birth month as well as sepsis in Table 2) included, TUNING The hyper-parameters of random forest and the artificial neural network are tuned for TABM-S (e.g., RF-500 and ANN-20 represent the random forest with 500 trees and the artificial neural network with two hidden layers of the size 20, respectively).

**Table 4:** Random Forest Variable Importance: Temperature Average for Birth Month, Sepsis Included.

Variable	Value	Rank
Gestational age	0.0476	**4**
Gestational age < 28 weeks	0.0100	32
Gestational age < 26 weeks	0.0102	31
Birth weight	0.0910	**1**
Birth weight Z-score	0.0907	**2**
Birth weight < 1000 g	0.0109	*30*
Birth weight < 750 g	0.0111	*27*
Small-for-gestational-age	0.0077	36
Sex (male)	0.0198	**10**
Birth-Year	0.0245	**6**
Birth-Month	0.0073	42
Birth-Season: Spring	0.0015	73
Birth-Season: Summer	0.0016	70
Birth-Season: Autumn	0.0015	72
Birth-Season: Winter	0.0014	74
Temperature average Year	0.0250	**5**
Temperature minimum Year	0.0244	**7**
Temperature maximum Year	0.0239	**8**
Temperature average 10-month before birth	0.0078	35
Temperature average 09-month before birth	0.0071	48
Temperature average 08-month before birth	0.0070	52
Temperature average 07-month before birth	0.0073	43
Temperature average 06-month before birth	0.0075	39
Temperature average 05-month before birth	0.0075	38
Temperature average 04-month before birth	0.0070	53
Temperature average 03-month before birth	0.0073	44
Temperature average 02-month before birth	0.0076	37
Temperature average 01-month before birth	0.0073	41
Temperature average 00-month before birth	0.0071	49
PM_10_ Year	0.0148	*21*
PM_10_ 10-month before birth	0.0074	40
PM_10_ 09-month before birth	0.0071	51
PM_10_ 08-month before birth	0.0069	56
PM_10_ 07-month before birth	0.0071	50
PM_10_ 06-month before birth	0.0072	45
PM_10_ 05-month before birth	0.0072	46
PM_10_ 04-month before birth	0.0070	55
PM_10_ 03-month before birth	0.0072	47
PM_10_ 02-month before birth	0.0070	54
PM_10_ 01-month before birth	0.0067	60
PM_10_ 00-month before birth	0.0069	57
Multiple pregnancy	0.0166	**13**
Multipara	0.0189	**11**
In vitro fertilization	0.0136	*23*
Gestational DM	0.0056	62
Overt DM	0.0015	71
Pregnancy-induced hypertension	0.0113	*26*
Chronic hypertension	0.0035	67
Chorioamnionitis	0.0163	**15**
PROM > 18 h	0.0149	*20*
Antenatal steroid	0.0125	*25*
Cesarean section	0.0156	*18*
Oligohydramnios	0.0110	*29*
Polyhydramnios	0.0024	69
Maternal age	0.0712	**3**
Maternal education	0.0159	*16*
Maternal citizenship	0.0067	59
Paternal education	0.0098	33
Paternal citizenship	0.0038	66
Unmarried	0.0044	64
Congenital infection	0.0034	68
1-min Apgar score ≤ 3	0.0150	*19*
5-min Apgar score < 7	0.0158	*17*
Neonatal resuscitation program	0.0045	63
Neonatal resuscitation program, intensive	0.0096	34
Blood gas pH < 7.0	0.0043	65
Blood gas base excess < -15	0.0060	61
Pulmonary hemorrhage	0.0134	*24*
Respiratory distress syndrome	0.0068	58
Surfactant use ≥ 2	0.0168	**12**
Patent ductus arteriosus treatment	0.0165	**14**
Patent ductus arteriosus ligation	0.0145	*22*
Air leak syndrome	0.0111	*28*
Sepsis	0.0237	**9**

Abbreviations: DM, Diabetes mellitus; PROM, Pre-labor rupture of membrane; PM_10_, particulate matter concentration.

The ranking of a top-15 (or top-30) predictor is highlighted in bold (or italic).

**Table 5:** Logistic Regression Variable Importance: Temperature Average for Birth Month, Sepsis Included.

Variable	Value	Rank	Raw
Gestational age	0.1647	*20*	0.1647
Gestational age < 28 weeks	0.2456	**13**	0.2456
Gestational age < 26 weeks	0.0655	34	−0.0655
Birth weight	0.0027	63	−0.0027
Birth weight Z-score	0.5274	**2**	0.5274
Birth weight < 1000 g	0.1619	*21*	0.1619
Birth weight < 750 g	0.2152	*17*	0.2152
Small-for-gestational-age	0.2596	**11**	0.2596
Sex (male)	0.3218	**7**	0.3218
Birth-Year	0.0019	65	−0.0019
Birth-Month	0.0051	57	0.0051
Birth-Season: Spring	0.0010	71	−0.0010
Birth-Season: Summer	0.0017	66	0.0017
Birth-Season: Autumn	0.0008	72	0.0008
Birth-Season: Winter	0.0016	67	−0.0016
Temperature average Year	0.0138	46	0.0138
Temperature minimum Year	0.2266	*16*	0.2266
Temperature maximum Year	0.2996	**8**	−0.2996
Temperature average 10-month before birth	0.0060	56	0.0060
Temperature average 09-month before birth	0.0042	59	−0.0042
Temperature average 08-month before birth	0.0014	68	−0.0014
Temperature average 07-month before birth	0.0043	58	0.0043
Temperature average 06-month before birth	0.0035	61	−0.0035
Temperature average 05-month before birth	0.0027	64	−0.0027
Temperature average 04-month before birth	0.0031	62	0.0031
Temperature average 03-month before birth	0.0007	73	0.0007
Temperature average 02-month before birth	0.0011	70	0.0011
Temperature average 01-month before birth	0.0012	69	0.0012
Temperature average 00-month before birth	0.0039	60	−0.0039
PM_10_ Year	0.0288	39	0.0288
PM_10_ 10-month before birth	0.0247	41	0.0247
PM_10_ 09-month before birth	0.0081	53	−0.0081
PM_10_ 08-month before birth	0.0097	50	−0.0097
PM_10_ 07-month before birth	0.0093	51	0.0093
PM_10_ 06-month before birth	0.0191	42	0.0191
PM_10_ 05-month before birth	0.0187	43	−0.0187
PM_10_ 04-month before birth	0.0136	47	−0.0136
PM_10_ 03-month before birth	0.0161	44	0.0161
PM_10_ 02-month before birth	0.0130	48	0.0130
PM_10_ 01-month before birth	0.0077	54	−0.0077
PM_10_ 00-month before birth	0.0005	74	−0.0005
Multiple pregnancy	0.1075	*28*	0.1075
Multipara	0.0753	33	0.0753
In vitro fertilization	0.2346	**14**	−0.2346
Gestational DM	0.5246	**3**	−0.5246
Overt DM	0.0807	32	−0.0807
Pregnancy-induced hypertension	0.1851	*19*	0.1851
Chronic hypertension	0.0076	55	0.0076
Chorioamnionitis	0.2802	**10**	−0.2802
PROM > 18 Hour	0.0859	*30*	0.0859
Antenatal Steroid	0.2334	**15**	0.2334
Cesarean section	0.1492	*24*	−0.1492
Oligohydramnios	0.1615	*22*	−0.1615
Polyhydramnios	0.0467	37	−0.0467
Maternal age	0.0088	52	0.0088
Maternal education	0.0601	35	−0.0601
Maternal citizenship	0.0128	49	−0.0128
Paternal education	0.1424	*25*	0.1424
Paternal citizenship	0.0141	45	0.0141
Unmarried	0.3626	**5**	0.3626
Congenital infection	0.0840	31	0.0840
1-min Apgar score ≤ 3	0.0927	*29*	0.0927
5-min Apgar score < 7	0.0470	36	0.0470
Neonatal resuscitation program	0.2107	*18*	−0.2107
Neonatal resuscitation program, intensive	0.1342	*26*	0.1342
Blood gas pH < 7.0	0.0374	38	−0.0374
Blood gas base excess < -15	0.2470	**12**	0.2470
Pulmonary hemorrhage	0.3511	**6**	0.3511
Respiratory distress syndrome	0.1317	*27*	0.1317
Surfactant use ≥ 2	0.0263	40	0.0263
Patent ductus arteriosus treatment	0.1543	*23*	0.1543
Patent ductus arteriosus litigation	0.4607	**4**	0.4607
Air leak syndrome	0.2935	**9**	0.2935
Sepsis	0.6989	**1**	0.6989

Abbreviations: DM, Diabetes mellitus; PROM, Pre-labor rupture of membrane; PM_10_, particulate matter concentration.

The ranking of a top-15 (or top-30) predictor is highlighted in bold (or italic).

## Discussion

Among the six prediction models for NEC, logistic regression and random forest had the best performances. According to random forest variable importance, major predictors of NEC included environmental factors (ambient birth year temperature), maternal factors (maternal age, multipara, multiple pregnancy, chorioamnionitis), and neonatal factors (GA, BW, male sex, sepsis, PDA).

This study confirmed that BW and GA were the main predictors of NEC. Our findings were consistent with the results of previous studies that revealed that lower BW and GA were the main risk factors for NEC^19,20^. Prematurity is well known to be the main cause of NEC. This can be explained by ischemic mucosal injury in the immature gut of preterm infants^21^. Recently, NEC has been considered to develop as multifactorial hits in the immature gut by both prenatal and postnatal factors. In addition, the gut microbiota in preterm infants is different from that in healthy term infants, and show a decreased diversity^22,23^. Moreover, prematurity reflects developmental changes in several organs other than in the gut, which increases the incidence of neonatal morbidity.

A unique finding of this study was that ambient temperature was associated with the incidence of NEC. The higher ambient temperature associated with NEC incidence may be influenced by environmental factors. Previous studies have reported that a high ambient temperature increases the risk of preterm birth^24–26^. Heat induces the production of proinflammatory cytokines such as interleukin (IL)-1, IL-6, and tumor necrosis factor, causing inflammatory processes at the maternal–fetal interface^27^. Furthermore, heat stress increases the production of oxytocin and prostaglandin, which are associated with uterine contractions and induce preterm labor^28,29^. It causes dehydration, resulting in decreases in maternal fluid levels, subsequently reducing fetal blood volume and leading to the production of pituitary hormones that provoke labor^30^.

Sepsis is one of the main predictors of NEC. Infection triggers inflammation in the immature gastrointestinal tract, which may contribute to NEC pathogenesis^31^. Recent findings have shown that preterm infants are exposed to a bacteria-rich environment in the neonatal intensive care unit and antibiotics that reduce the diversity of the gut microbiome^32^. Toll-like receptor 4 (TLR4) is a pathogen recognition molecule that recognizes bacterial endotoxins such as lipopolysaccharides and induces inflammation^33^. This TLR4-mediated bacterial signaling leads to increased mucosal injury and reduced mucosal repair, resulting in mucosal defects in which bacteria can translocate through the circulation^34–36^. At this stage, bacteria lead to the inhibition of vasodilator expression, thus decreasing intestinal perfusion, which results in tissue necrosis of the gut^37^.

In this study, chorioamnionitis was found to be a predictor of NEC. There have been debates regarding prenatal infection or inflammation and its effects on NEC. Some studies reported no association, but others demonstrated that chorioamnionitis was associated with preterm birth, and it was also associated with inflammation and infection in infants during perinatal periods^38–40^. A meta-analysis by Been et al. revealed that chorioamnionitis is significantly associated with NEC^41^. Our findings are consistent with the results of these studies. Gastrointestinal inflammatory markers were increased in preterm infants exposed to chorioamnionitis, reflecting the proinflammatory state of the gut after birth^42^. The gut microbiome reflects amniotic fluid with chorioamnionitis^43^. In this condition, preterm infants may have disturbed barrier function, which would increase the susceptibility of the gut to secondary hits, such as sepsis and circulatory instability, leading to an increased incidence of NEC^41^.

In this study, multiparity was significantly associated with NEC. Lee et al. reported similar results in VLBW infants^40^. This finding may explain why the infant can be affected by maternal parity, exposure to maternal stress factors from recurrent pregnancy, oxidative stress, and passive transfer of immunomodulators that change the gut microbiota of neonates.

There are some limitations to this study. First, address information was not provided in the Korean Neonatal Network (KNN) database; hence, national averages were taken for PM_10_ and temperature variables in this study. More specific information on these predictors would improve the validity of research in this direction. Second, this study did not consider the possible mediating effects of the various predictors. Third, this study did not focus on examining the possible mechanisms between major predictors and NEC. Fourth, this study did not include indoor factors that could be major predictors of NEC. Fifth, it was beyond the scope of this study to compare various re-sampling approaches regarding class imbalance, i.e., the proportion of NEC was only 6.8%. Under-sampling involves the reduction of the majority class for the balance, whereas over-sampling involves the expansion of the minority class for the goal. For example, a recent study compared the performance measures of four machine learning models in the cases of under-sampling and over-sampling for the prediction of cardiovascular disease^44^. Few studies are available, and further investigation is needed on this topic. Sixth, maternal age, GA, BW, BW Z-score and environmental predictors were not normalized in order to keep their full information. Using different rescaling methods for these continuous predictors (e.g., normalization) and comparing their results would make a valuable contribution for this line of research. Seventh, this study followed existing literature ^49,53,54^ to focus on top-10 predictors in terms of random forest variable importance. However, it needs to be noted that there has been no consensus on the threshold of major predictors in terms of random forest variable importance. Eighth, this study focused on random forest variable importance instead of logistic regression variable importance. Logistic regression performed as good as did the random forest in this study. But logistic regression requires an unrealistic assumption of *ceteris paribus*, i.e., “all the other variables staying constant.” For this reason, we used random forest variable importance for evaluating the importance ranking of a major predictor and univariate analysis for testing the direction of association between NEC and the predictor. Some predictors ranked within the top 15 in the random forest but out of the top 30 in logistic regression, i.e., BW (1st vs. 63rd), maternal age (3rd vs. 52nd), average birth year temperature (5th vs. 56th), birth year (6th vs. 65th), primipara (11thvs. 33rd) and surfactant use (12th vs. 40th). Little literature is available and more examination is needed on comparing the variable importance of various statistical approaches.

To the best of our knowledge, the performance of the random forest in this study (the area under the receiver operating characteristic curve of 0.72) is among the highest in this line of research. NEC is strongly associated with birth year temperature, as well as maternal and neonatal predictors.

## Methods

### Participants and variables

The data consisted of 10,353 VLBW infants from the KNN database from January 2013 to December 2017. The KNN started in April 2013 as a national prospective cohort registry of VLBW infants admitted or transferred to neonatal intensive care units across South Korea (it covers 74 neonatal intensive care units now). It collects perinatal and neonatal data of VLBW infants based on a standardized operating procedure^45^.

The dependent variable was NEC, with binary categories (no, yes). The following 47 perinatal predictors were considered (43 of them had binary categories): sex, birth-year (categorical: 2013, 2014, 2015, 2016, 2017), birth-month, birth-season (spring, summer, autumn, winter), multiple pregnancy, in vitro fertilization, gestational diabetes mellitus, overt diabetes mellitus, pregnancy-induced hypertension, chronic hypertension, histologic chorioamnionitis, pre-labor rupture of membranes > 18 h, antenatal steroid, cesarean section, oligohydramnios, polyhydramnios, maternal age (years), primipara, maternal education (categorical: elementary, junior high, senior high, college or higher), maternal citizenship, paternal education (categorical: elementary, junior high, senior high, college or higher), paternal citizenship, marital status, congenital infection, 1-min Apgar score ≤ 3, 5-min Apgar score < 7, neonatal resuscitation program, intensive neonatal resuscitation (intubation, chest compression or medications), initial blood gas pH < 7.0, initial blood gas base excess < -15, pulmonary hemorrhage, respiratory distress syndrome, surfactant use ≥ 2, PDA treatment (medical or surgical), PDA ligation, air leak syndrome, GA, GA < 28 weeks, GA < 26 weeks, BW, BW Z-score, BW < 1,000 g, BW < 750 g, SGA, and sepsis. The following 26 environmental predictors were also included: PM_10_ for birth year, PM_10_ for each month during pregnancy, average ambient temperature for birth year, minimum ambient temperature for birth year, maximum ambient temperature for birth year, and average ambient temperature for each month during pregnancy. PM_10_ and ambient temperature data were obtained from the Korea Meteorological Administration (KMA) (PM_10_ https://data.kma.go.kr/data/climate/selectDustRltmList.do?pgmNo=68; temperature https://web.kma.go.kr/weather/climate/past_cal.jsp). According to the KMA, PM_10_ denotes the concentration of particles with diameters of 10 µm or less, whereas ambient temperature represents the overall temperature of the outdoor air surrounding people.

NEC was diagnosed according to the modified Bell’s staging criteria (≥ Stage II)^46^. Gestational diabetes mellitus was defined as any degree of glucose intolerance with the onset or first recognition during pregnancy. Pregnancy-induced hypertension was defined as hypertension with onset in the latter part of pregnancy (> 20 weeks’ gestation), followed by normalization of blood pressure postpartum. Chorioamnionitis was defined as histologic chorioamnionitis^47^. Oligohydramnios (or polyhydramnios) was defined as an amniotic fluid index of < 5 cm (or > 24 cm). Small-for-GA was defined as BW below the 10^th^ percentile, according to the Fenton growth chart^48^.

### Statistical analysis

Artificial neural networks, decision trees, logistic regression, naïve Bayes, random forests, and support vector machines were used for predicting NEC^49–54^. The following default parameters were adopted for convenience: The splitting criterion was GINI, the max depth was not determined and the number of trees was 1000 in the random forest; the radial basis function kernel was employed in the support vector machine; and the limited memory Broyden–Fletcher–Goldfarb–Shanno algorithm served for the optimization of the artificial neural network. Data on 10,353 observations with full information were divided into training and validation sets in a 70:30 ratio. Accuracy, which is the ratio of correct predictions among 3,106 observations, was employed as the standard for validating the models. Random forest variable importance, the contribution of a certain variable to the performance (GINI) of the random forest, was used to examine the major predictors of NEC in VLBW infants, including environmental factors. The random split and analysis were repeated 50 times, and the average was used for external validation^55,56^. Different seed numbers were used for different runs but the default parameters stayed the same throughout the random splits and analyses. R-Studio 1.3.959 (R-Studio Inc.: Boston, United States) was employed for the analysis from August 1, 2021 to September to 30, 2021.

### Ethical statement

The KNN registry was approved by the institutional review board (IRB) at each participating hospital (IRB No. of Korea University Anam Hospital: 2013AN0115). Informed consent was obtained from the parent(s) of each infant registered in the KNN. All methods were carried out in accordance with the IRB-approved protocol and in compliance with relevant guidelines and regulations.

The names of the IRB of the KNN participating hospitals are as follows: The Institutional Review Board of Gachon University Gil Medical Center, The Catholic University of Korea Bucheon ST. Mary’s Hospital, The Catholic University of Korea Seoul ST. Mary’s Hospital, The Catholic University of Korea ST. Vincent’s Hospital, The Catholic University of Korea Yeouido ST. Mary’s Hospital, The Catholic University of Korea Uijeongbu ST. Mary’s Hospital, Gangnam Severance Hospital, Kyung Hee University Hospital at Gangdong, GangNeung Asan Hospital, Kangbuk Samsung Hospital, Kangwon National University Hospital, Konkuk University Medical Center, Konyang University Hospital, Kyungpook National University Hospital, Gyeongsang National University Hospital, Kyung Hee University Medical Center, Keimyung University Dongsan Medical Center, Korea University Guro Hospital, Korea University Ansan Hospital, Korea University Anam Hospital, and Kosin University Gospel Hospital, National Health Insurance Service Iilsan Hospital, Daegu Catholic University Medical Center, Dongguk University Ilsan Hospital, Dong-A University Hospital, Seoul Metropolitan Government-Seoul National University Boramae Medical Center, Pusan National University Hospital, Busan ST. Mary’s Hospital, Seoul National University Bundang Hospital, Samsung Medical Center, Samsung Changwon Medical Center, Seoul National University Hospital, Asan Medical Center, Sungae Hospital, Severance Hospital, Soonchunhyang University Hospital Bucheon, Soonchunhyang University Hospital Seoul, Soonchunhyang University Hospital Cheonan, Ajou University Hospital, Pusan National University Children’s Hospital, Yeungnam University Hospital, Ulsan University Hospital, Wonkwang University School of Medicine & Hospital, Wonju Severance Christian Hospital, Eulji University Hospital, Eulji General Hospital, Ewha Womans University Medical Center, Inje University Busan Paik Hospital, Inje University Sanggye Paik Hospital, Inje University Ilsan Paik Hospital, Inje University Haeundae Paik Hospital, Inha University Hospital, Chonnam National University Hospital, Chonbuk National University Hospital, Cheil General Hospital & Women’s Healthcare Center, Jeju National University Hospital, Chosun University Hospital, Chung-Ang University Hospital, CHA Gangnam Medical Center, CHA University, CHA Bundang Medical Center, CHA University, Chungnam National University Hospital, Chungbuk National University, Kyungpook National University Chilgok Hospital, Kangnam Sacred Heart Hospital, Kangdong Sacred Heart Hospital, Hanyang University Guri Hospital, and Hanyang University Medical Center.

### Ethics approval and consent to participate

Data collection was approved by the institutional review board of each hospital participating in KNN (2013AN0115). Informed consent was obtained from the parents (s) of each infant registered in the KNN.

## Data Availability

The code used in this study is available from the corresponding author upon reasonable request. The data presented in this study are not publicly available. However, the data are available from the corresponding author upon reasonable request and with permission from the Korean Neonatal Network and the Korea Centers for Disease Control and Prevention.

## References

[1] Neu J, Walker WA (2011). Necrotizing enterocolitis. N. Engl. J. Med..

[2] Stoll BJ (2010). Neonatal outcomes of extremely preterm infants from the NICHD Neonatal Research Network. Pediatrics.

[3] Horbar JD (2012). Mortality and neonatal morbidity among infants 501 to 1500 grams from 2000 to 2009. Pediatrics.

[4] Clark RH (2012). Characteristics of patients who die of necrotizing enterocolitis. J. Perinatol..

[5] Hintz SR (2005). Neurodevelopmental and growth outcomes of extremely low birth weight infants after necrotizing enterocolitis. Pediatrics.

[6] Rees CM, Pierro A, Eaton S (2007). Neurodevelopmental outcomes of neonates with medically and surgically treated necrotizing enterocolitis. Arch. Dis. Child. Fetal Neonatal Ed..

[7] Shah DK (2008). Adverse neurodevelopment in preterm infants with postnatal sepsis or necrotizing enterocolitis is mediated by white matter abnormalities on magnetic resonance imaging at term. J. Pediatr..

[8] Claud EC, Walker WA (2008). Bacterial colonization, probiotics, and necrotizing enterocolitis. J. Clin. Gastroenterol..

[9] Blau J (2011). Transfusion-related acute gut injury: necrotizing enterocolitis in very low birth weight neonates after packed red blood cell transfusion. J. Pediatr..

[10] Dollberg S, Lusky A, Reichman B (2005). Patent ductus arteriosus, indomethacin and necrotizing enterocolitis in very low birth weight infants: a population-based study. J. Pediatr. Gastroenterol. Nutr..

[11] Lambert DK (2012). Fulminant necrotizing enterocolitis in a multihospital healthcare system. J. Perinatol..

[12] Moss RL (2008). Clinical parameters do not adequately predict outcome in necrotizing enterocolitis: A multi-institutional study. J. Perinatol..

[13] Sankaran K (2004). Variations in incidence of necrotizing enterocolitis in Canadian neonatal intensive care units. J. Pediatr. Gastroenterol. Nutr..

[14] Stritzke AI, Smyth J, Synnes A, Lee SK, Shah PS (2013). Transfusion-associated necrotising enterocolitis in neonates. Arch. Dis. Child. Fetal Neonatal Ed..

[15] Snyder CL, Hall M, Sharma V, St Peter SD (2010). Seasonal variation in the incidence of necrotizing enterocolitis. Pediatr. Surg. Int..

[16] Javidi D, Wang Z, Rajasekaran S, Hussain N (2021). Temporal and seasonal variations in incidence of stage II and III NEC-a 28-year epidemiologic study from tertiary NICUs in Connecticut, USA. J. Perinatol..

[17] Murphy T (2019). Necrotizing enterocolitis and spontaneous intestinal perforation: A spatiotemporal case cluster analysis. Pediatr. Qual. Saf..

[18] Ahle M, Drott P, Andersson RE (2013). Epidemiology and trends of necrotizing enterocolitis in Sweden: 1987–2009. Pediatrics.

[19] Samuels N, van de Graaf RA, de Jonge RCJ, Reiss IKM, Vermeulen MJ (2017). Risk factors for necrotizing enterocolitis in neonates: A systematic review of prognostic studies. BMC Pediatr..

[20] Gephart, S. M., McGrath, J. M., Effken, J. A. & Halpern, M. D. Necrotizing enterocolitis risk: state of the science. *Adv. Neonatal Care* **12**, 77–87; quiz 88–79 (2012). 10.1097/ANC.0b013e31824cee94 PMC3357630 22469959

[21] Crissinger KD (1994). Regulation of hemodynamics and oxygenation in developing intestine: insight into the pathogenesis of necrotizing enterocolitis. Acta Paediatr. Suppl..

[22] Korpela K (2018). Intestinal microbiota development and gestational age in preterm neonates. Sci. Rep..

[23] Wandro, S. *et al.* The microbiome and metabolome of preterm infant stool are personalized and not driven by health outcomes, including necrotizing enterocolitis and late-onset sepsis. *mSphere* **3** (2018). 10.1128/mSphere.00104-18 PMC5990886 29875143

[24] Basu R, Chen H, Li DK, Avalos LA (2017). The impact of maternal factors on the association between temperature and preterm delivery. Environ. Res..

[25] Ha S (2017). Ambient temperature and early delivery of singleton pregnancies. Environ. Health Perspect..

[26] Walfisch A, Kabakov E, Friger M, Sheiner E (2017). Trends, seasonality and effect of ambient temperature on preterm delivery. J. Matern. Fetal Neonatal Med..

[27] Lee SE, Park IS, Romero R, Yoon BH (2009). Amniotic fluid prostaglandin F2 increases even in sterile amniotic fluid and is an independent predictor of impending delivery in preterm premature rupture of membranes. J. Matern. Fetal Neonatal Med..

[28] Kelly, A. J., Kavanagh, J. & Thomas, J. Vaginal prostaglandin (PGE2 and PGF2a) for induction of labour at term. *Cochrane Database Syst. Rev.* (2), CD003101 (2001). 10.1002/14651858.CD003101 11406078

[29] Stan, C. M., Boulvain, M., Pfister, R. & Hirsbrunner-Almagbaly, P. Hydration for treatment of preterm labour. *Cochrane Database Syst. Rev.* (11), CD003096 (2013). 10.1002/14651858.CD003096.pub2 PMC11751767 24190310

[30] Lee SS, Kwon HS, Choi HM (2008). Evaluation of preterm delivery between 32–33 weeks of gestation. J. Korean Med. Sci..

[31] Neish AS (2004). Molecular aspects of intestinal epithelial cell-bacterial interactions that determine the development of intestinal inflammation. Inflamm. Bowel Dis..

[32] Hackam D, Caplan M (2018). Necrotizing enterocolitis: pathophysiology from a historical context. Semin. Pediatr. Surg..

[33] Hargreaves DC, Medzhitov R (2005). Innate sensors of microbial infection. J. Clin. Immunol..

[34] Neal MD (2013). A critical role for TLR4 induction of autophagy in the regulation of enterocyte migration and the pathogenesis of necrotizing enterocolitis. J. Immunol..

[35] Lu, P. *et al.* Animal models of gastrointestinal and liver diseases. Animal models of necrotizing enterocolitis: Pathophysiology, translational relevance, and challenges. *Am. J. Physiol. Gastrointest. Liver Physiol.* **306**, G917-G928 (2014). 10.1152/ajpgi.00422.2013 PMC4042110 24763555

[36] Hackam DJ, Sodhi CP (2018). Toll-like receptor-mediated intestinal inflammatory imbalance in the pathogenesis of necrotizing enterocolitis. Cell. Mol. Gastroenterol. Hepatol..

[37] Yazji, I. *et al.* Endothelial TLR4 activation impairs intestinal microcirculatory perfusion in necrotizing enterocolitis via eNOS-NO-nitrite signaling. *Proc. Natl Acad. Sci. U. S. A.* **110**, 9451–9456 (2013). 10.1073/pnas.1219997110 PMC3677476 23650378

[38] Lau J (2005). Chorioamnionitis with a fetal inflammatory response is associated with higher neonatal mortality, morbidity, and resource use than chorioamnionitis displaying a maternal inflammatory response only. Am. J. Obstet. Gynecol..

[39] Romero R (2007). The role of inflammation and infection in preterm birth. Semin. Reprod. Med..

[40] Lee JY (2017). Maternal and placental risk factors for developing necrotizing enterocolitis in very preterm infants. Pediatr. Neonatol..

[41] Been JV, Lievense S, Zimmermann LJ, Kramer BW, Wolfs TG (2013). Chorioamnionitis as a risk factor for necrotizing enterocolitis: A systematic review and meta-analysis. J. Pediatr..

[42] Arnon S, Grigg J, Silverman M (1993). Association between pulmonary and gastric inflammatory cells on the first day of life in preterm infants. Pediatr. Pulmonol..

[43] Miralles R (2005). Relationship between antenatal inflammation and antenatal infection identified by detection of microbial genes by polymerase chain reaction. Pediatr. Res..

[44] Oh T (2022). Machine learning-based diagnosis and risk factor analysis of cardiocerebrovascular disease based on KNHANES. Sci. Rep..

[45] Chang YS, Ahn SY, Park WS (2013). The establishment of the Korean neonatal network (KNN). Neonatal. Med..

[46] Bell, M. J. *et al.* Neonatal necrotizing enterocolitis. Therapeutic decisions based upon clinical staging. *Ann. Surg.* **187**, 1–7 (1978). 10.1097/00000658-197801000-00001 PMC1396409 413500

[47] Yoon BH (1995). Amniotic fluid interleukin-6: a sensitive test for antenatal diagnosis of acute inflammatory lesions of preterm placenta and prediction of perinatal morbidity. Am. J. Obstet. Gynecol..

[48] Fenton TR, Kim JH (2013). A systematic review and meta-analysis to revise the Fenton growth chart for preterm infants. BMC Pediatr..

[49] Lee KS, Ahn KH (2019). Artificial neural network analysis of spontaneous preterm labor and birth and its major determinants. J. Korean Med. Sci..

[50] Lee KS, Song IS, Kim ES, Ahn KH (2020). Determinants of spontaneous preterm labor and birth including gastroesophageal reflux disease and periodontitis. J. Korean Med. Sci..

[51] Lee, K. S. & Ahn, K. H. Application of artificial intelligence in early diagnosis of spontaneous preterm labor and birth. *Diagnostics (Basel)* **10**, 733 (2020). 10.3390/diagnostics10090733 PMC7555184 32971981

[52] Lee, K. S. *et al.* Association of preterm birth with depression and particulate matter: machine learning analysis using national health insurance data. *Diagnostics (Basel)* **11**, 555 (2021). 10.3390/diagnostics11030555 PMC8003604 33808913

[53] Ahn, K. H. *et al.* Predictors of Newborn's weight for height: A machine learning study using nationwide multicenter ultrasound data. *Diagnostics (Basel)* **11**, 1280 (2021). 10.3390/diagnostics11071280 PMC8304217 34359366

[54] Lee KS, Kim ES, Kim DY, Song IS, Ahn KH (2021). Association of gastroesophageal reflux disease with preterm birth: Machine learning analysis. J. Korean Med. Sci..

[55] Park EK (2019). Machine learning approaches to radiogenomics of breast cancer using low-dose perfusion computed tomography: Predicting prognostic biomarkers and molecular subtypes. Sci. Rep..

[56] Lee JY (2022). Radiomic machine learning for predicting prognostic biomarkers and molecular subtypes of breast cancer using tumor heterogeneity and angiogenesis properties on MRI. Eur. Radiol..

